# Clinicopathological Investigation and PCR-Based Molecular Detection of *Toxoplasma gondii* Infection in a Red Kangaroo (*Osphranter rufus*): A Case Report

**DOI:** 10.3390/vetsci13080794

**Published:** 2026-08-09

**Authors:** Tayyaba Ashiq, Kexin Yu, Xiaoxiang Pan, Babar Maqbool, Shuying Zhong, Zhaofeng Hou, Yanhong Wang, Penggang Liu

**Affiliations:** 1 College of Veterinary Medicine, Yangzhou University, Yangzhou 225009, China; ashiqtayyaba564@gmail.com (T.A.); 15051720837@163.com (K.Y.); m17802524998@163.com (X.P.); zhongshuying2024@163.com (S.Z.); zfhou@yzu.edu.cn (Z.H.); wyh7405@163.com (Y.W.); 2 Babar Maqbool Department of Veterinary Medicine, Faculty of Veterinary and Animal Sciences, University of Agriculture, Dera Ismail Khan 29111, Pakistan; babermaqbool1@gmail.com; 3 Joint International Research Laboratory of Agriculture and Agri-Product Safety of the Ministry of Education of China, Yangzhou University, Yangzhou 225009, China

**Keywords:** *Toxoplasma gondii*, kangaroo, PCR, hematology, histopathology

## Abstract

Toxoplasmosis is a zoonotic disease caused by the intracellular protozoan parasite *Toxoplasma gondii*, with marsupials recognized as particularly vulnerable hosts. This is a case report of the integrated investigation of fatal illness in a captive red kangaroo (*Osphranter rufus*). Multisystemic lesions with hemorrhages and congestion of the lungs, liver, spleen, kidneys, heart, and intestines were detected during gross necropsy. Hematological analysis demonstrated leukopenia (2.90 × 10^9^/L), neutropenia (1.19 × 10^9^/L), lymphopenia (1.53 × 10^9^/L), and increased hematocrit (61.2%). The serum alanine aminotransferase level was very high (216 U/L), and urea nitrogen and creatinine were elevated, indicating involvement of the liver and kidneys. A bacteriological investigation based on the sequences of 16S rDNA revealed the presence of *Neisseria weaveri* from the heart and *Staphylococcus arlettae* from the kidney; no *Salmonella* spp. or *Clostridium perfringens* were isolated. The DNA of *T. gondii* was detected by PCR in various tissues, including spleen (low-level) and lung (high-level). Pathohistological examination of the spleen demonstrated lymphoid depletion and inflammatory infiltration without tissue cysts, tachyzoites, granulomatous inflammation, or multifocal necrosis. Characteristic lesions are absent, and there is no way to definitively attribute mortality to fatal toxoplasmosis, as the lung had the strongest PCR signal. Collectively, these results support PCR-based molecular detection of disseminated *T. gondii* DNA and emphasize the necessity of comprehensive histopathological correlation with all PCR-positive organs in future investigations.

## Simple Summary

This report provides an integrated diagnostic investigation of a PCR-positive captive red kangaroo by combining gross pathology, hematology, serum biochemistry, bacteriology, histopathology, and molecular testing. The results add to the sparse literature on toxoplasmosis in macropods and highlight the importance of a multi-disciplinary approach in diagnosing wildlife diseases. Taken together, these findings support PCR-based molecular detection of disseminated *T. gondii* DNA while also highlighting the diagnostic challenge associated with the absence of pathognomonic histopathological lesions in susceptible marsupials.

## 1. Introduction

*Toxoplasma gondii* is an obligatory intracellular apicomplexan parasite that has a global distribution and is known to pose serious clinical and economic consequences in both animals and humans [1]. The definitive hosts are felids, which shed environmentally resistant oocysts, which infect a wide variety of intermediate hosts, such as domestic and wildlife species, including kangaroos [2]. Clinical disease severity depends on the species, and also on the host’s immunity status and the strain of the parasite [3]. Marsupials in general, and macropods, especially kangaroos (*Osphranter rufus*), are highly vulnerable to acute, systemic toxoplasmosis [4]. In contrast to eutherian mammals that develop subclinical infection, macropods frequently evolve to severe disseminated disease with high morbidity and mortality. There have also been reports of clinical manifestations of respiratory distress, neurological dysfunction, lethargy, anorexia, and sudden death, which often have not been preceded by any clear ante-mortem conditions. Such non-discriminating conditions pose diagnostic challenges, especially in the zoological, conservation, and rehabilitation settings [5]. Diagnosis of toxoplasmosis has to be carried out in a multidisciplinary manner. Hematologic and biochemical tests may indicate systemic inflammation and organ involvement; histopathological findings include inflammatory infiltration, lymphoid depletion, granulomatous inflammation, multifocal necrosis, and tachyzoites and tissue cysts, whereas the distribution and severity of lesions vary among susceptible species [6]. *Toxoplasma gondii* DNA can be sensitively and specifically detected using molecular techniques, specifically polymerase chain reaction (PCR), which is sensitive to *T. gondii* sequences and can be detected in infected tissues [7].

Despite this susceptibility, reports of toxoplasmosis in macropods combined with molecular and histopathological findings remain limited. Therefore, the present case study aims to describe the molecular, histopathological, and hematological investigation of *Toxoplasma gondii* infection in a kangaroo (*Osphranter rufus*) and highlights the importance of early molecular diagnosis of toxoplasmosis in the highly vulnerable exotic species.

## 2. Case History and Clinical Examination

On 7 October 2024, a 6-month-old female captive red kangaroo (*Osphranter rufus*) maintained in a zoological park in Jiangsu Province, China, died suddenly and was submitted to the Animal Clinical Diagnostic Laboratory, Veterinary Teaching Hospital, Yangzhou University, for a comprehensive etiological study. kangaroo pellets, fresh alfalfa hay, and vegetables were provided with ad libitum water.

About 10 days before death, the animal became lethargic and inappetent with mild respiratory problems (tachypnea and occasional open-mouth breathing). The animal’s condition worsened despite supportive care, such as fluid therapy and antibiotics (enrofloxacin, 5 mg/kg SID for 3 days). Within 24 h of death, the animal was very weak, had lost his appetite, and was in severe respiratory distress. During this period, no other kangaroos in the collection exhibited clinical signs.

Immediately after death, blood samples and other tissues were collected, such as the heart, liver, spleen, lungs, kidneys, and intestinal contents, and transported under refrigerated conditions to be analyzed in laboratories.

## 3. Materials and Methods

### 3.1. Routine and Biochemical Examination

Tissue samples (heart, liver, spleen, lung, kidney, lymph nodes, and intestinal contents) were taken from representative locations at post-mortem examination and sent to the Animal Clinical Diagnostic Laboratory at the Veterinary Teaching Hospital of Yangzhou University in China. Blood samples were collected within 10 min of the animal’s death and placed at 4 °C until further processing.

An automated hematology analyzer (Sysmex XT-2000iV, Kobe, Japan, Sysmex Corporation) was used to analyze the whole blood in terms of RBC count and WBC count, hematocrit, and differential leukocyte count. Biochemical parameters of serum, such as ALT, total protein, albumin, blood urea nitrogen (BUN), and creatinine, were analyzed by a biochemical autoanalyzer (Sysmex XT-2000iV, Kobe, Japan, Sysmex Corporation). Published data of captive red [7] were used to determine reference intervals for hematological and biochemical parameters.

### 3.2. Bacterial Isolation and Identification

Heart, liver, spleen, lung, and kidney samples were inoculated on sheep blood agar and MacConkey agar and incubated aerobically at 37 °C for 24–48 h. A culture on sheep blood agar under anaerobic conditions was performed at 37 °C for 24 h to identify *Clostridium perfringens*. Bacterial identification was carried out by amplification of the 16S rDNA gene fragment using primers F (5′-TGCTTGCGGAACTTTGACTC-3′) and R (5′-CCACACTGATGTCGTTTCTTG-3′), resulting in an amplicon of 571 bp. The purified PCR products were sequenced and queried in the NCBI GenBank database using the BLAST tool to identify the sequence to a taxonomic level.

The sequence of the purified PCR products was commercially sequenced (Sangon Biotech, Shanghai, China), and the sequences were compared to the NCBI GenBank database using BLAST for taxonomic identification.

### 3.3. Salmonella Detection

The liver tissues and intestinal contents were pre-enriched with buffered peptone water, and the selective enrichment was followed by plating on *Salmonella* chromogenic agar and MacConkey agar. The cultures were incubated at 37 °C and studied for characteristic colonies.

### 3.4. Molecular Detection of Toxoplasma gondii

Tissue DNA samples were subjected to genomic DNA extraction using a commercial genomic DNA extraction kit (Qiagen, Hilden, Germany). Spectrophotometric analysis was used for determining the concentration and purity of DNA before downstream analysis. *Toxoplasma gondii* was detected using the conventional PCR method based on the B1 gene, with the primers previously described by Burg et al. [8] that amplify a 196 bp fragment. The assays contained appropriate positive and negative controls to ensure the analytical validity. The products were amplified, then separated on an agarose gel and visualized with an ultraviolet transilluminator. The presence of a band of 196 bp was considered as the presence of *T. gondii* DNA in the samples analyzed.

### 3.5. Histopathological Examination (H&E Staining)

The histopathological investigation was restricted to spleen tissue, as lung, kidney, and liver tissues were not available for examination. Tissue of the spleen were fixed using 4% neutral buffered formalin and subjected to normal procedures and embedded in paraffin, sectioned at 4–5 µm, and stained with hematoxylin and eosin (H&E). Slides were examined under light microscopy to analyze the structural and cellular changes.

## 4. Results

### 4.1. Gross Necropsy Findings

**Figure 1:**
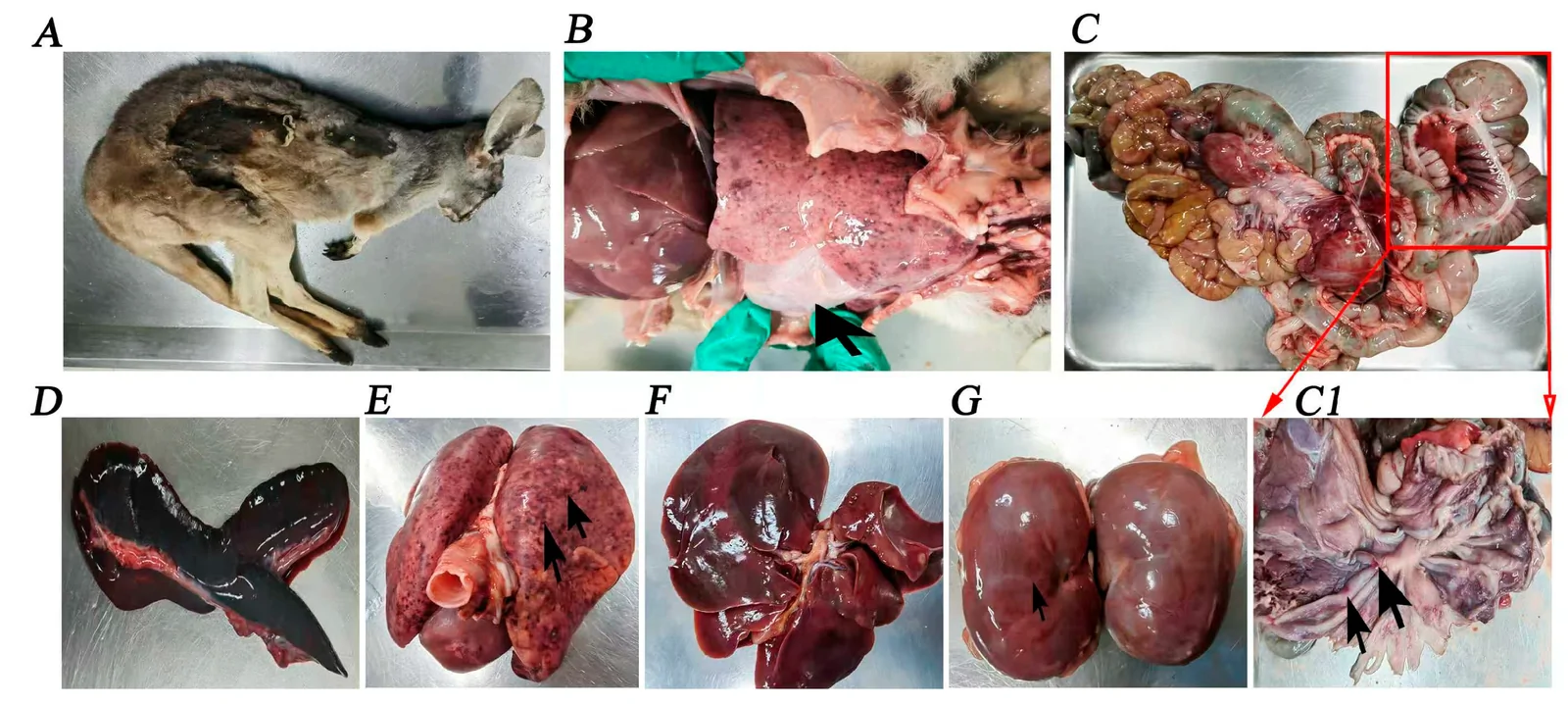
(**A**): Kangaroo; (**B**): Heart; (**C**): Intestine; (**C1**) is the intestinal dissection arrow indicating the bleeding point, (**D**): the spleen. (**E**): The lungs’ arrow indicates the bleeding point; (**F**): Liver; (**G**): Kidney. The arrow indicates the bleeding point.

The carcass showed irregular dark ecchymotic patches across the body surface (Figure 1A),which represented systemic circulatory disturbance. The thorax and abdomen were opened, and a diffuse congestion and hemorrhagic lesions were observed in several visceral organs. The heart showed myocardial hyperemia and subepicardial hemorrhage in a few areas (Figure 1B, arrow). Intestinal tract exhibited Severe hemorrhagic enteritis (longitudinal incision of the intestine, arrows) with the intestinal wall swelling and extensive petechial and ecchymotic hemorrhages clearly exposed after longitudinal incision of the intestine (Figure 1C,C1). The spleen was highly congested and swollen (Figure 1D), consistent with splenic hyperemia and possible hemorrhagic infarction. Lung parenchyma exhibited diffuse pulmonary congestion, multifocal pulmonary hemorrhage, and pulmonary consolidation (Figure 1E, arrows). The kidney and liver were bilaterally swollen with a dark reddish color and discrete superficial hemorrhagic spots (Figure 1F,G). There were also a few lesions of the distal digestive tract with a submucosal hemorrhagic nature.

Gross postmortem examination indicated systemic congestion and multifocal hemorrhagic lesions in the heart, lungs, spleen, kidney, and intestinal tract, suggesting a systemic hemorrhagic pathological process in the affected kangaroo.

### 4.2. Blood Routine and Biochemical Testing

Hematological and biochemical analysis revealed multiple abnormalities, summarised in Table 1 [9].

**Table 1:** Hematological and biochemical parameters.

Parameter	Value	Reference Range
WBC (×10^9^/L)	2.90	4.00–19.00
Neutrophils (×10^9^/L)	1.19	1.72–10.61
Lymphocytes (×10^9^/L)	1.53	2.68–11.54
Hematocrit (%)	61.2	21.0–38.0
ALT (U/L)	216	5–28
Total Protein (g/L)	59.6	57–81
Albumin (g/L)	24.4	20–60
BUN (mmol/L)	8.39	1.8–7.1
Creatinine (µmol/L)	211	53–159

WBC count (2.90 × 10^9^/L), neutrophil, and lymphocyte values were below the reference range (4.00–19.00 × 10^9^/L). Elevated levels of hematocrit, ALT, BUN, and creatinine were observed.

### 4.3. Bacteriological Culture and Identification

**Figure 2:**
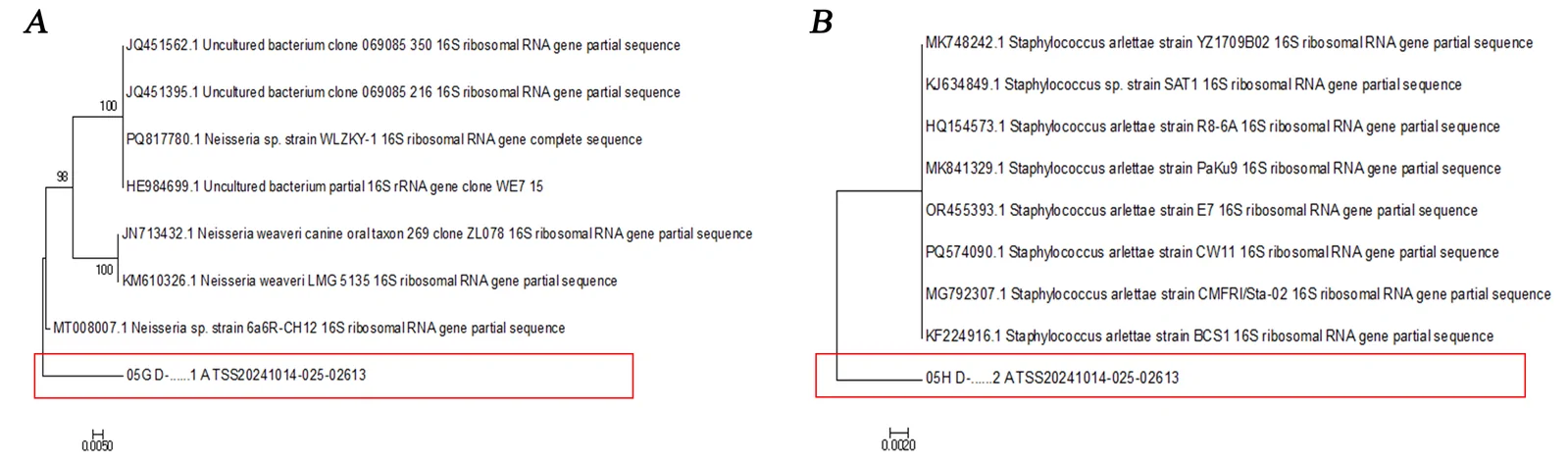
Anaerobic culture demonstrated the presence of bacterial growth from the heart (**A**) and kidney (**B**).

**Figure 3:**
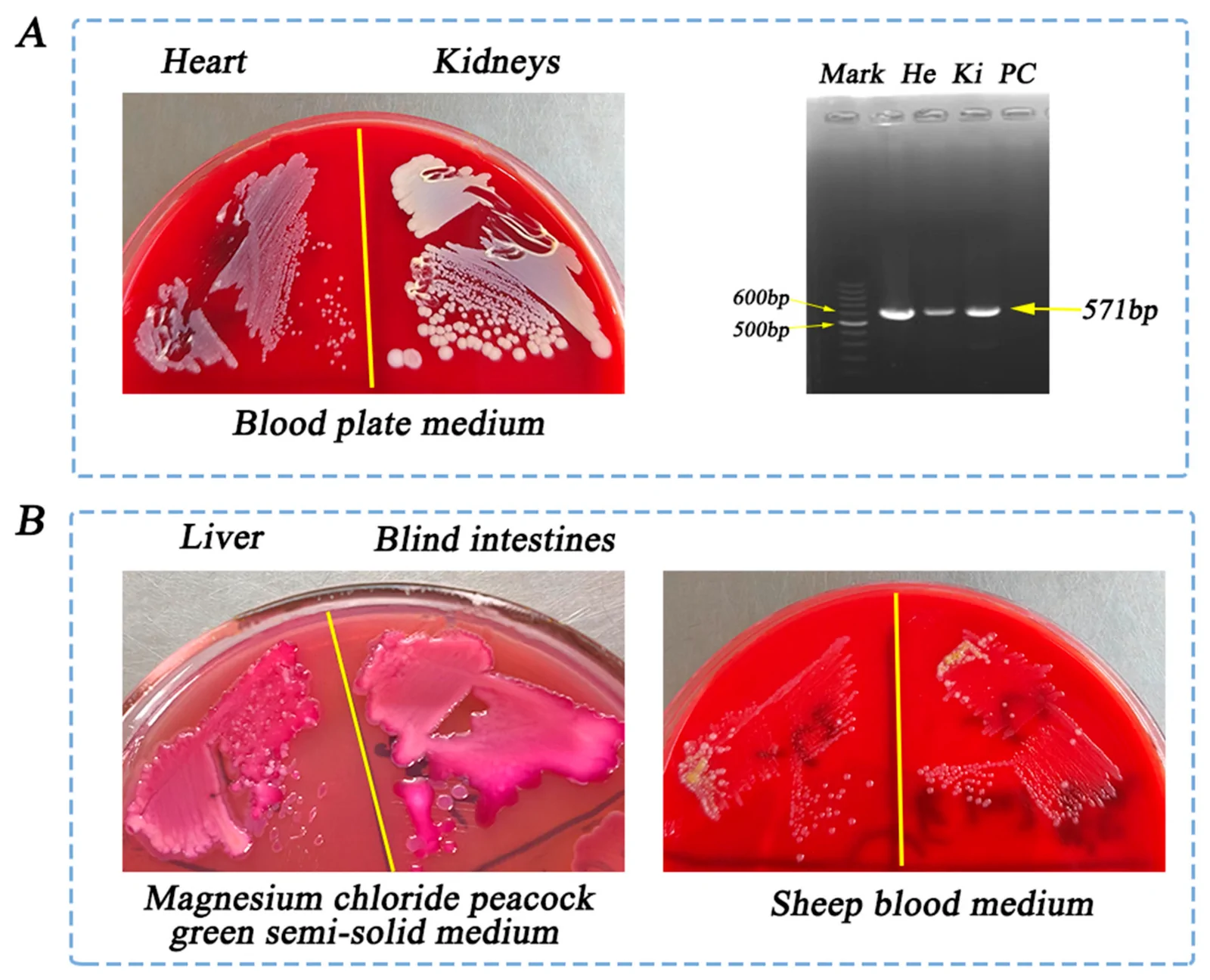
Figure (**A**) shows colonies of *Neisseria weaveri* on blood agar from heart samples and *Staphylococcus arlettae* from kidney samples. Figure (**B**) shows no colonies of *Salmonella* appeared on MacConkey agar (left dish), while no hemolytic colonies of *Clostridium* appeared on blood agar from liver and intestinal samples, respectively, less contributor in the death of the animal.

Anaerobic culture demonstrated the presence of bacterial growth from the heart and kidney (Figure 2A,B). 16S rDNA sequencing revealed the presence of *Neisseria weaveri* (98% identity, MT008007.1, JN713432.1, and KM610326.1) from the heart. *Staphylococcus arlettae* (100% identity, MK748242.1, KJ634849.1, HQ154573.1, and so on) from the kidney (Figure 3A). Other tissues demonstrated the absence of growth. The hemolytic colonies that were indicative of *Clostridium perfringens* were not identified in anaerobic culture. No *Salmonella* spp. was recovered (Figure 3B). These bacterial findings suggest these organisms are less likely to be primary cause of death.

### 4.4. Molecular Detection of Toxoplasma gondii

**Figure 4:**
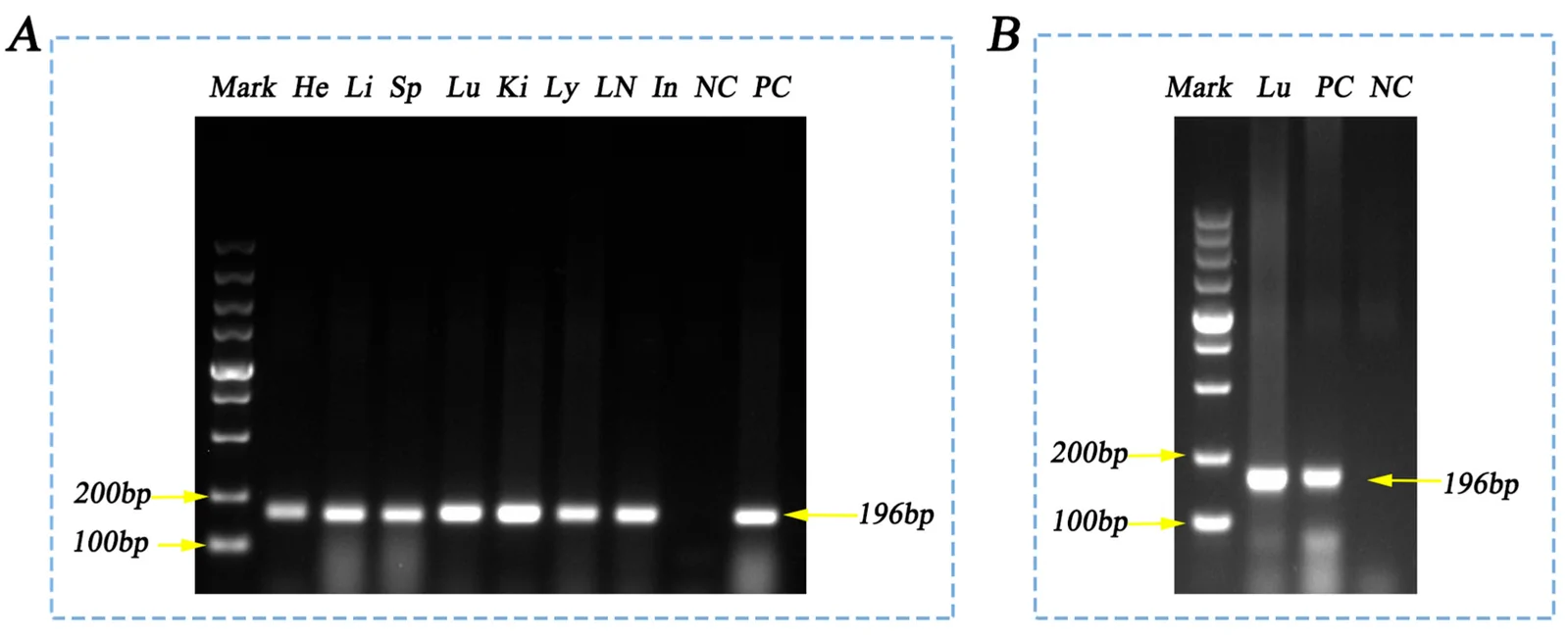
Figure (**A**) shows PCR amplification for different samples of kangaroo, including heart, liver, spleen, lungs, kidney, lymph nodes, and intestines, showing positive 196 bp bands for *Toxoplasma gondii* while in the lungs tissue sample, a very strong 196 bp specific band for *Toxoplasma gondii* was shown Figure (**B**).

On subsequent tests (on the 7 and 10 October 2024), PCR amplification gave specific bands for *Toxoplasma gondii* for various tissues of animals, along with positive and negative controls (Figure 4A), but amplification was most clearly observed in the lung tissue sample (Figure 4B).The orignal uncropeped gel images provided in Figure S1. In the spleen tissue, there was weak amplification. The positive control exhibited expected amplification, whereas the negative control showed no band.

These findings indicate PCR-based molecular detection of *T. gondii* in multiple tissues and support systemic distribution of parasite DNA in the examined animal. In particular, the high amplification in the pulmonary tissue is in agreement with the well-documented tropism of *T. gondii* for the lungs during acute systemic infection in macropods [10].

### 4.5. Histopathological Findings

**Figure 5:**
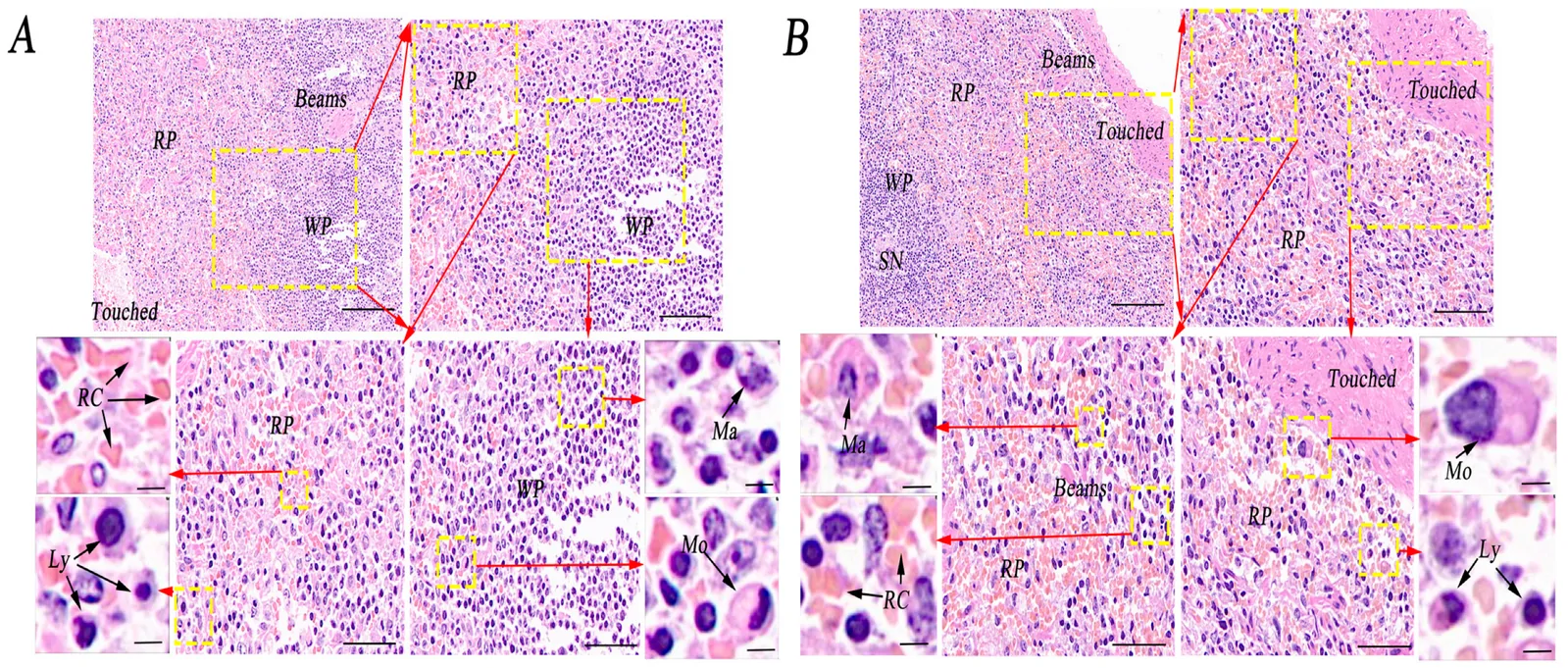
H&E, ×400 magnification Scale Bar: 50 µm Figure (**A**) shows the main spleen trunk, while figure (**B**) shows the accessory spleen/splenunculus. Both the red pulp and white pulp of the spleen showed a significant increase in infiltration of inflammatory cells. (RP: red pulp; WP: white pulp; RC: red blood cell; Ly: lymphocytes; Mo: monocyte; Ma: macrophage).

Under microscopy (H&E, ×400 magnification), histopathological examinations of the spleen showed severe depletion of the lymphoid tissue (white pulp) in the spleen with lower lymphocyte density. The red pulp exhibited marked vascular congestion, with marked accumulation of erythrocytes and inflammatory cells (mainly mononuclear cells, neutrophils, and lymphocytes). These results are indicative of an acute inflammatory process. (Figure 5A) shows the histology of the main spleen trunk, and (Figure 5B) shows the histology of the accessory spleen or splenunculus. Critically, granulomatous inflammation, tissue cysts, foci of necrosis, or tachyzoites were not observed in any splenic section. These findings limit the confirmation of fatal toxoplasmosis.

## 5. Discussion

Macropods are known to be highly susceptible and can develop acute systemic toxoplasmosis after exposure to an environmentally contaminated oocysts, unlike many other eutherian mammals, which can develop subclinical infection [11]. In other studies, disseminated infection and sudden death have been reported in captive kangaroos with severe clinical disease, highlighting the susceptibility of these species to disseminated infection [7]. Red kangaroos (*Osphranter rufus*) have been reported as a frequent host of *T. gondii* infection, which can cause rapid clinical deterioration and death [12]. Kangaroos and other Australian marsupials are known to be highly susceptible to *T. gondii* infection, which results in clinical illness and, subsequently, death [13].

In this case, the kangaroo presented as dyspneic and lethargic before death. Multisystemic hemorrhagic and congestive lesions in the lungs, liver, spleen, kidneys, intestine, and heart were observed during gross necropsy, which is indicative of severe systemic disease. However, these lesions were suggestive of multisystem involvement, though not pathognomonic for toxoplasmosis, and necessitated comprehensive laboratory investigation.

Hematological examination demonstrated leukopenia (2.90 × 10^9^/L), neutropenia (1.19 × 10^9^/L), lymphopenia (1.53 × 10^9^/L), and increased hematocrit (61.2%). Serum biochemical parameters showed that alanine aminotransferase (ALT), blood urea nitrogen (BUN), and creatinine levels were elevated. High ALT levels may be a sign of hepatocellular damage from systemic *Toxoplasma gondii* infection, while elevated BUN and creatinine levels may be due to renal failure, dehydration, or impaired renal perfusion during serious systemic disease. These findings are consistent with systemic disease but not specific for toxoplasmosis. In fatal cases of kangaroos and other marsupials, toxoplasmosis has been reported with similar changes, but these changes are not regarded as diagnostic of toxoplasmosis [9].

The parasite was detected by PCR in several tissues, which was strongly positive in the lung and weakly positive in the spleen. In acute toxoplasmosis of macropods, tachyzoites are present in large numbers in the lungs of the animals, and inflammatory lesions are often seen [14]. PCR detection, however, only shows the presence of parasite DNA and does not show definitive causality. Therefore, clinical, gross pathological, clinicopathological, and histopathological findings should be interpreted along with molecular findings.

Splenic lymphoid depletion, vascular congestion, and inflammatory cell infiltration were found in the spleen by histopathological examination. Fatal toxoplasmosis in captive marsupials typically presents as lesions in the lungs, liver, heart, and other internal organs; severity and extent of lesions vary [10]. Clinical history, gross pathology, histopathology, and ancillary laboratory tests are all important, and integration is essential for the diagnosis of toxoplasmosis in zoo animals, rather than a single diagnostic finding [15]. Consequently, the absence of pathognomonic histopathological lesions combined with the inability to examine lung histopathology and other PCR-positive organs limits the ability to definitively attribute mortality to toxoplasmosis.

Furthermore, the bacteria found, *Neisseria weaveri* and *Staphylococcus arlettae*, were identified using 16S rDNA sequencing. These organisms, however, were isolated from one organ, but the other organs were negative. Furthermore, two important bacterial pathogens of marsupials, *Salmonella* spp. and *Clostridium perfringens*, were not detected. All of these observations together indicate that the bacterial isolates were probably secondary or incidental infections, and not the primary cause of death.

Collectively, Clinicopathological, bacteriological and molecular findings are all supportive of PCR-based molecular detection of disseminated *T. gondii* DNA but do not constitute definitive evidence of fatal toxoplasmosis. Results should be considered suggestive of systemic *T. gondii* infection with the understanding that further confirmation of causality with immunohistochemistry, in situ hybridization, or histopathological examination of the lung should be sought for future investigation.

## 6. Conclusions

This case report describes PCR-based molecular detection of disseminated *T. gondii* DNA in a 6-month-old female captive red kangaroo (*Osphranter rufus*) supported by gross necropsy findings, hematological and biochemical abnormalities, bacteriological studies, and limited splenic histopathology.

Three levels of evidence must be clearly differentiated. First, multisystemic areas of hemorrhage and congestion were noted grossly in the lungs, liver, spleen, kidneys, heart, and intestine. Second, *T. gondii* DNA was detected from various tissues by PCR, with the highest amplification examined in the lungs.

Third, definitive etiological confirmation remained unattained, as histopathological examination of the spleen revealed lymphoid depletion, vascular congestion, and infiltration with inflammatory cells. Importantly, no tissue cysts, tachyzoites, foci of necrosis, or granulomatous inflammation were observed. No typical histopathological findings were observed, preventing the definitive pathological confirmation of toxoplasmosis as the cause of death. Therefore, findings provide supportive evidence of disseminated *T. gondii* DNA, rather than pathological confirmation of fatal toxoplasmosis.

This report has highlighted the benefits of combining gross pathology, clinicopathological assessment, bacteriological tests, molecular detection, and histopathology for the investigation of fatal infectious diseases in highly susceptible marsupials. However, it also underscores the comprehensive histopathological analysis of all PCR-positive organs and the use of confirmatory methods such as in situ hybridization or immunohistochemistry in future cases to provide definitive causality.

## Data Availability

The original contributions presented in this study are included in the article/supplementary material. Further inquiries can be directed to the corresponding author.

## Supplementary Materials

The following supporting information can be downloaded at: https://www.mdpi.com/article/10.3390/vetsci13080794/s1, File S1: PCR Original image.
